# KRIBB11: A Promising Drug that Promotes Microglial Process Elongation and Suppresses Neuroinflammation

**DOI:** 10.3389/fphar.2022.857081

**Published:** 2022-03-18

**Authors:** Jianbin Su, Zhihua Dou, Hongxiang Hong, Feng Xu, Xu Lu, Qun Lu, Ting Ye, Chao Huang

**Affiliations:** ^1^ Department of Endocrinology, Affiliated Hospital 2 of Nantong University, First People’s Hospital of Nantong City, Nantong, China; ^2^ Department of Pharmacy, Nantong Third Hospital Affiliated to Nantong University, Nantong, China; ^3^ Department of Spine Surgery, Affiliated Hospital 2 of Nantong University, First People’s Hospital of Nantong City, Nantong, China; ^4^ Department of Pharmacology, School of Pharmacy, Nantong University, Nantong, China

**Keywords:** KRIBB11, microglia, process elongation, Akt, neuroinflammation

## Abstract

Microglia are key components of the central innate immune system. The over-activation of microglia, which occurs in nervous system disorders, is usually accompanied with retractions of their ramified processes. Reversing of microglial process retraction is a potential strategy for the prevention of neuroinflammation. Our previous studies have reported some endogenous molecules and drugs that can promote microglial process elongation at conditions *in vitro* and *in vivo*, such as butyrate and β-hydroxybutyrate, sulforaphane, and diallyl disulfide. Here, reported another compound that can promote microglial process elongation. We found that KRIBB11, a compound which has been reported to suppress nitric oxide production in microglia, induced significant elongations of the processes in microglia in cultured and *in vivo* conditions in a reversible manner. KRIBB11 pretreatment also prevented lipopolysaccharide (LPS)-induced shortenings of microglial process in cultured conditions and *in vivo* conditions, inflammatory responses in primary cultured microglia and the prefrontal cortex, and depression-like behaviors in mice. Mechanistic studies revealed that KRIBB11 incubation up-regulated phospho-Akt in cultured microglia and Akt inhibition blocked the pro-elongation effect of KRIBB11 on microglial process in cultured conditions and *in vivo* conditions, suggesting that the regulatory effect of KRIBB11 is Akt-dependent. Akt inhibition was also found to abrogate the preventive effect of KRIBB11 on LPS-induced inflammatory responses in primary cultured microglia and prefrontal cortexes as well as LPS-induced depression-like behaviors in mice. Collectively, our findings demonstrated that KRIBB11 is a novel compound that can prevent microglial activation and neuroinflammation-associated behavioral deficits possibly through inducing the Akt-mediated elongation of microglial process.

## Introduction

Microglia are innate immune cells in the central nervous system which make great contributions to brain homeostasis regulation (Hammond et al., 2021). In physiological conditions, the microglia usually present morphologies with ramified processes which are known to be in charge of synaptic pruning, synaptic remodeling, and debris clearance (Harry, 2013; Bar and Barak, 2019; Gosselin, 2020). In pathological conditions, the microglia would be over-activated and show amoeboid morphologies and typical pro-inflammatory phenotypes, which subsequently mediate neuronal damage and behavioral deficits (Hanlon et al., 2019; Matta et al., 2020).

The induction of amoeboid morphology has been shown to be accompanied with microglial over-activation and the progression of neuroinflammatory responses in the brain (Reichert and Rotshenker, 2019; Takagi et al., 2019), while the ramified morphology shows positive correlations with anti-neuroinflammation and neuroprotection (Vinet et al., 2012; Yang R. et al., 2019). Studies by McWhorter et al. had provided direct evidence for the correlations between immune cell process elongation and anti-neuroinflammation: they found that promotion of macrophage elongation by control of cell shape directly increases the expression of anti-inflammatory mediators and meanwhile reduces the secretion of pro-inflammatory cytokines (Neubrand et al., 2014). In recent studies, we and others had reported that molecules like butyrate (Wang et al., 2018), adenosine (Matyash et al., 2017), β-hydroxybutyrate (Huang et al., 2018), diallyl disulfide (Xu et al., 2020), and sulforaphane (Wu et al., 2019) simultaneously promote microglial process elongation and transform the microglia into an anti-inflammatory phenotype, which was manifested by the decreased expression of pro-inflammatory cytokines, such as interleukin-1β (IL-1β) and IL-6, and the increased expression of anti-inflammatory mediators, such as IL-4 and IL-10. These results demonstrated that promotion of microglial process elongation may be a potential strategy for the prevention of neuroinflammation, and searching drugs that induce microglial process elongation may be of great significance for the development of strategies that can prevent microglial activation and neuroinflammation-associated behavioral deficits.

KRIBB11 is a compound that is preliminarily developed as an inhibitor of heat shock factor 1 (HSF1) (Yoon et al., 2011). As HSF1 is highly correlated with heat shock response and tumor pathogenesis, the KRIBB11 is usually used to investigate the correlation between HSF1 and cancers: the inhibition of HSF1 activity by KRIBB11 treatment can suppress the growth of tumor cells (Fok et al., 2018; Lee et al., 2021). One of our previous studies had reported that KRIBB11 treatment, possibly by inhibiting the activity of HSF1, prevents the expression or production of inducible nitric oxide synthase (iNOS) and nitric oxide (NO) in lipopolysaccharide (LPS)-stimulated primary cultured microglia or in brain tissues in mice treated with a toxic dosage of LPS by reducing the binding of nuclear factor-κB (NF-κB) and signal transducer and activator of transcription 1 (STAT1) to the DNA elements in iNOS gene promoters (Huang et al., 2015). As increased iNOS and NO are known to represent a high level of neuroinflammatory response (Ivan et al., 2021; Wang T. et al., 2021), this finding demonstrated that the KRIBB11 may possess an ability to suppress microglial activation.

In further analysis, we found that KRIBB11 not only reduced the expression or production of iNOS and NO but also induced dramatic elongations of the processes in the primary cultured microglia. Considering the negative correlations between microglial process elongation and the neuroinflammatory phenotypes (Huang et al., 2018; Wang et al., 2018; Wu et al., 2019; Xu et al., 2020), we assumed that the KRIBB11 treatment may prevent neuroinflammation in a manner that is dependent on its pro-elongation effect on microglial process. In this study, we designed a series of experiments to examine this hypothesis, and found that the KRIBB11 treatment induced remarkable elongations of the processes in microglia in cultured and *in vivo* conditions by activating protein kinase B (Akt), a molecule that has been proved to mediate the rearrangement of cellular actin filaments and microtubule cytoskeletons (Seiberlich et al., 2015; Antonosante et al., 2020). KRIBB11 pretreatment was also found to prevent LPS-induced shortenings of microglial process in cultured and *in vivo* conditions, as well as LPS-induced neuroinflammatory responses and depression-like behaviors. These results not only identified a candidate for the regulation of microglial process dynamics, but also revealed that the KRIBB11 could be developed as a drug for the suppression of neuroinflammation and neuroinflammation-associated behavioral abnormalities.

## Materials and Methods

### Materials

LPS (*Escherichia coli*, serotype 0111: B4, #L2630) and poly-L-lysine (#25988-63-0) were purchased from Sigma (Saint Louis, MO, United States) and Santa Cruz Biotechonology (Santa Cruz, CA, United States), respectively. Both Hoechst 33258 (#HY-15558) and KRIBB11 (#HY-100872) are the products of MedChem Express (Princeton, NJ, United States). Antibodies against protein kinase B (Akt, #4691), phospho-Akt (#4060), and glyceraldehyde-3-phosphate dehydrogenase (GAPDH, #5174) are the products of Cell Signaling Technology (Beverly, MA, United States). The antibody against Iba-1 (#ab178846) was purchased from Abcam (Cambridge, MA, United States). Both LY294002 (#440202) and VIII (#124018) were purchased from Calbiochem (San Diego, CA, United States). Dulbecco’s Modified Eagle’s Medium/F12 (DMEM/F12) was obtained from Biotium and Gibco Invitrogen Corporation. Other agents were purchased from commercial suppliers. Stocked drugs solutions were stored at −20°C and diluted to the final concentration immediately before use. The final concentration of dimethyl sulfoxide (DMSO) is <0.05%. No toxic effect of DMSO was observed.

### Animals

Eight-week old male C57BL6/J mice were purchased from Beijing Vital River Laboratory Animal Technology Co., Ltd. (Beijing, China) and were housed five per cage under standard conditions (12-h light/dark cycle, 07:00‒19:00 light, 23 ± 1°C of temperature, and 55 ± 10% of humidity) for 1 week before use with free access to food and water. Animal experiments were conducted in accordance with internationally accepted guidelines for the use of animals in toxicology as adopted by the Society of Toxicology in 1999) and were approved by the University Animal Ethics Committee of Nantong University (Permit Number: 2110836).

### Preparation of Primary Cultured Microglia

Primary cultured microglia were prepared according to our previous studies (Huang et al., 2018). Brain cortexes obtained from newborn mice (day 0–1) were removed and digested with 0.125% trypsin at 37°C for 15 min. Followed by trituration and centrifugation at 118 g for 5 min, mixed cells were re-suspended and plated on poly-L-lysine (1 mg/ml)-coated culture flasks. The individual cell suspensions were cultured in DMEM/F12 supplement with 10% FBS and 1% penicillin-streptomycin (100 U/mL). This medium was replaced every 3 days. After 10 days, the mixed cells were shaken gently 2 h at 37°C and the supernatants were collected and plated on the new poly-L-lysine-coated culture flasks. Cells were maintained in an incubator (37°C) containing 95% air and 5% CO_2_. The purity of microglia (>99%) was identified by the anti-Iba-1 antibody.

### Cell Viability Assay

The microglial viability was measured using an MTT Cell Proliferation and Cytotoxicity Assay Kit purchased from Bi Yuntian Biological Technology Institution (Shanghai, China). The methylthiazolyldiphenyl-tetrazolium bromide (5 mg/ml) was firstly dissolved in prepared MTT solutions and kept at −20°C. After washing, 20 μL of MTT solutions were incubated with the microglia in at 37°C for 4 h. The blue crystals were dissolved in formazan-dissolved solutions. The absorbance was read at 570 nm.

### Experimental Arrangement

**FIGURE 1:**
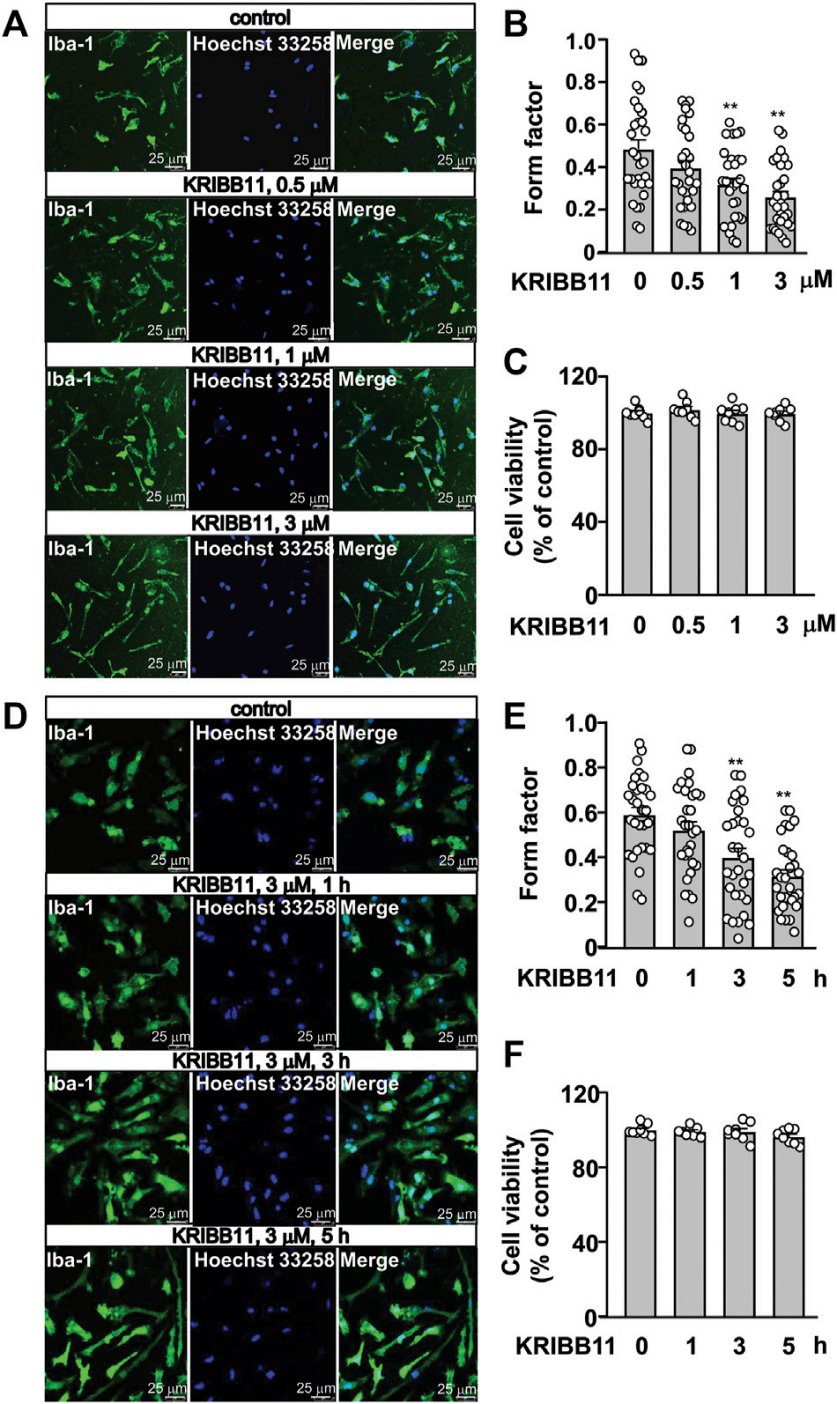
Dose- and time-dependent effects of KRIBB11 on the processes in primary cultured microglia. **(A,B)** Representative images **(A)** and quantitative analysis **(B)** showed that KRIBB11 induced significant elongations of the processes in primary cultured microglia at the dosages of 1 and 3 μM (*p < 0.05 or **p < 0.01 vs. vehicle). **(C)** Quantitative analysis showed the effect of KRIBB11 (0.5, 1, 3 μM; 5 h) on microglial viability (*n* = 8). **(D,E)** Representative images **(D)** and quantitative analysis **(E)** showed the pro-elongation effect of KRIBB11 on primary cultured microglial process at time points ranging from 1 to 5 h (**p < 0.01 vs. vehicle). **(F)** Quantitative analysis showed the effect of KRIBB11 at different time points (3 μM; 1, 3, and 5 h) on microglial viability (*n* = 8). For cell shape investigation, 30 cells per condition were selected in three independent experiments. Scale bars: 25 μm. Data are shown as mean ± SEM.

**FIGURE 2:**
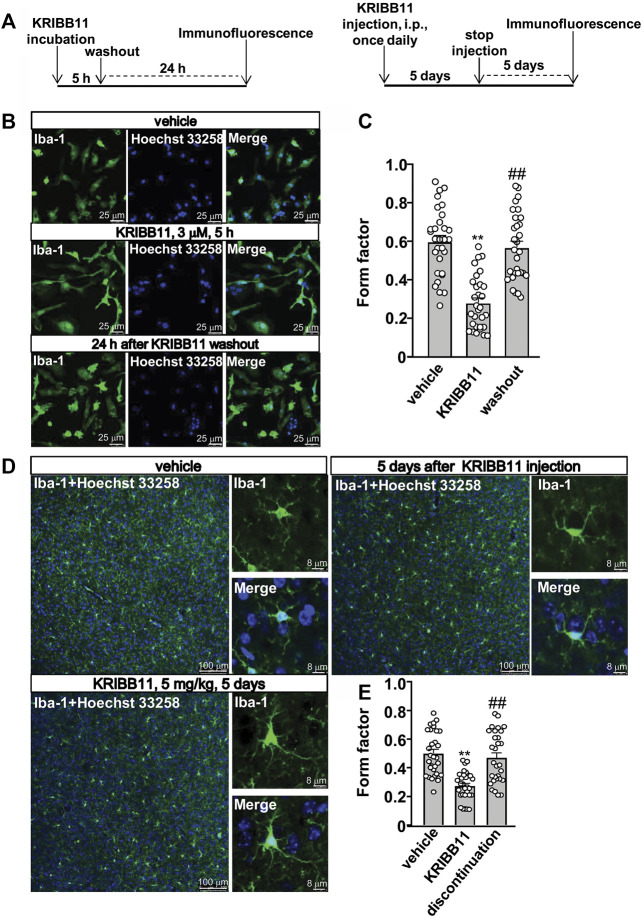
Reversible effects of KRIBB11 on microglial process at conditions *in vitro* and *in vivo*. **(A)** A schematic diagram showing the timeline for the evaluation of the reversal effect of KRIBB11 treatment on microglial process elongation at conditions *in vitro* (left) and *in vivo* (right). **(B,C)** Representative images **(B)** and quantitative analysis **(C)** showed the effect of KRIBB11 treatment (3 μM, 5 h) and washout on primary cultured microglial process (**p < 0.01 vs. vehicle, ##*p* < 0.01 vs. KRIBB11). **(D,E)** Representative images **(D)** and quantitative analysis **(E)** showed the changes in microglial process in the dorsolateral prefrontal cortex before, during, and 5 days after KRIBB11 treatment (5 mg/kg; **p < 0.01 vs. vehicle, ##*p* < 0.01 vs. KRIBB11). For cell shape investigation at conditions *in vitro* and *in vivo*, 30 cells per condition were analyzed in three independent experiments. Scale bars for *in vitro* analysis is 25 μm, and scale bars for *in vivo* analysis in the low and high magnification images are 100 and 8 μm, respectively. Data are shown as mean ± SEM.

In the dose-dependent experiment in Figure 1A, the primary cultured microglia were incubated with different dosages of KRIBB11, including 0.5, 1, and 3 μM, for 5 h. In the time-dependent experiment in Figure 1D, the primary cultured microglia were incubated with 3 μM of KRIBB11 for different time period, including 1, 3, and 5 h. In the reversible experiments, the primary cultured microglia incubated with 5 h of KRIBB11 (3 μM) were collected 24 h after KRIBB11 washout, and the dorsolateral prefrontal cortexes in mice injected with 5 days of KRIBB11 (once daily, i.p., 5 mg/kg) were collected 5 days after the discontinuation of KRIBB11 injection (Figure 2A).

To evaluate the functional consequence of KRIBB11 pretreatment on microglial morphology, neuroinflammatory response, and behavioral abnormalities, the primary cultured microglia or the experimental mice were allocated into vehicle, KRIBB11 (*in vitro* studies: 1 h of pretreatment, 3 μM; *in vivo* studies: 5 days of pretreatment, 5 mg/kg), LPS treatment (*in vitro* studies: 1 μg/ml, 8 h; *in vivo* studies: 100 μg/kg, 5 h), KRIBB11 + LPS groups (morphology analysis: *n* = 30, in each group; inflammation analysis: *n* = 8, in each group; behavioral assays: *n* = 10, in each group).

To evaluate the role of Akt in the pro-elongation effect of KRIBB11 on microglial process and inflammatory response, the cultured microglia were allocated into six groups, including the vehicle, KRIBB11, LY294002, KRIBB11 + LY294002, VIII, KRIBB11 + VIII groups. During this experiment, the inhibitor of the Akt signaling, LY294002 (20 μM) or VIII (10 μM), was pre-incubated 30 min before KRIBB11 treatment. To evaluate the role of Akt in the regulatory effect of KRIBB11 on microglial process, inflammatory response, or behavioral abnormalities at normal and inflammatory conditions, the cultured microglia or the experimental mice were allocated into eight groups, including the vehicle, KRIBB11, LY294002, KRIBB11 + LY294002, vehicle + LPS, KRIBB11 + LPS, LY294002 + LPS, KRIBB11 + LY294002 + LPS groups. For *in vivo* studies, the LY294002 was infused into the lateral ventricle during KRIBB11 treatment; for *in vitro* studies, the LY294002 was given before KRIBB11 treatment, and cells were collected 8 h after LPS treatment. Behavioral assays were conducted 5 h after the single LPS injection, and the fresh dorsolateral prefrontal cortexes were collected immediately after the discontinuation of behavioral assays. The dosages of LPS and KRIBB11 used in animal studies were selected according to our previous studies (Huang et al., 2015; Huang et al., 2018). Both KRIBB11 and LPS were administered intraperitoneally in a volume of 10 ml/kg.

### Immunofluorescence and Form Factor Analysis

This experiment was conducted according to our previous studies (Huang et al., 2018). The experimental mice were anaesthetized with pentobarbital sodium and perfused transcardially with 4% paraformaldehyde in PBS, and their brains were frozen and sectioned with a cryostat at 20 μm. The brain sections in a 24-well plate were permeabilized with 0.3% Triton X-100 for 30 min and incubated with 3% bovine serum albumin-PBS solution for another 30 min at room temperature, followed by a further incubation in a PBS solution containing 0.3% Triton X-100, 1% BSA, and anti-Iba-1 antibody (1:500) overnight at 4°C. After that, the sections were incubated in fluorescein isothiocyanate-labeled horse anti-rabbit IgG (1:50) for 2 h at room temperature, followed by a further Hoechst 33258 incubation (10 min). The final cover-slipped slides were examined using an Olympus FV-500 confocal microscope and camera (Tokyo, Japan). The changes in process length were analyzed using the form factor. Each image was processed by the median filter at a radius of eight pixels under a black and white threshold image using ImageJ software. Cell surroundings drawn by the wand tracing tool were used to determine the area and the perimeter of each cell. Cells touching the borders of the image were excluded from the statistical analysis. The form factor was calculated using the formula 4π*area/(perimeter)^2^ (Wilms et al., 1997). A value close to one corresponds to round cells and approaching 0 indicate highly ramified cells. At least 80 cells per condition were analyzed in at least three independent experiments.

### Real-Time Polymerase Chain Reaction

The total RNA in the primary cultured microglia and dorsolateral prefrontal cortex in mice with or without vehicle, KRIBB11, LPS, and/or LY294002 treatment was extracted by using an RNeasy mini kit according to manufacturer’s instructions (Qiagen, GmbH, Hilden, Germany). The first-strand of cDNA was generated by using a reverse transcription system (Promega, Madison, WI, United States), and the real-time PCR was conducted with a reaction system which contained 1 × Faststart SYBR Green Master Mix (Roche Molecular Biochemicals), 2 μL of diluted cDNA, 2 mM MgCl_2_, and 0.5 μM of primers. Primers for IL-1β, IL-6, IL-4, IL-10, arginase-1, and CD206 are cited as follows (Xu et al., 2020; Gu et al., 2021; Wang K. et al., 2021): IL-β: 5′-TGG​AAA​AGC​GGT​TTG​TCT​TC-3’ (F), 5′-TAC​CAG​TTG​GGG​AAC​TCT​GC-3′ (R); IL-6: 5′-AGA​GAT​ACA​AAG​AAA​TGA​TGG​A-3′ (F), 5′-AGC​TAT​GGT​ACT​CCA​CAA​GAC​CA-3′ (R); IL-4: 5′-CAG​CTA​GTT​GTC​ATC​CTG​CTC​TTC-3′ (F), 5′-GCC​GAT​GAT​CTC​TCT​CAA​GTG​A-3′ (R); IL-10: 5′-GGC​AGA​GAA​CCA​TGG​CCC​AGA​A-3′ (F), 5′-AAT​CGA​TGA​CAG​CGC​CTC​AGC​C-3′ (R); arginase-1: 5′-CTT​GCG​AGA​CGT​AGA​CCC​TG-3′ (F), 5′-TGA​GTT​CCG​AAG​CAA​GCC​AA-3’ (R); CD206: 5′-CTT​CGG​GCC​TTT​GGA​ATA​AT-3′ (F), 5′-TAG​AAG​AGC​CCT​TGG​GTT​GA-3′ (R). The primers for the GAPDH are 5′-GGC​CTT​CCG​TGT​TCC​TAC-3′ (F) and 5′-TGT​CAT​CAT​ATC​TGG​CAG​GTT-3′ (R). The PCR products were detected by monitoring the increase in intensity of fluorescence emitted by the double-stranded DNA-binding dye SYBR Green. An analysis of gene expression was performed by using the −ΔΔCt method, and their values were normalized to GAPDH.

### Western Blot

This experiment was performed according to our previous studies (Huang et al., 2018). Briefly, the cell lysates were firstly centrifuged at 12,000 g for 16 min, and the supernatants were harvested. After being denatured, 30 μg of protein was separated on 10% SDS/PAGE gels and then transferred to nitrocellulose membranes (Bio-Rad, Hercules, CA, United States). After being blocked with 5% nonfat dried milk powder/Tris-buffered saline Tween-20 (TBST) for 1 h, the nitrocellulose membranes were probed with the anti-phospho-Akt (1:1,000), anti-Akt (1:1,000), or anti-GAPDH (1:10000) antibody overnight at 4°C. The primary antibody was then removed by washing the membranes three times in TBST. Membranes were further incubated for 2 h at room temperature with IRDye 680-labeled secondary antibodies (1:3,000–1:5,000). The immunoblots were visualized using the Odyssey CLx western blot detection system, and their band densities were quantified using ImageJ software.

### Tail Suspension Test and Forced Swimming Test

The TST and FST were performed according to our previous studies (Gu et al., 2021). For the TST, the experimental mice were suspended by taping their tail (1 cm from top) to an elevated (50 cm above ground) wooden bar and applied plastic tubes over tails to avoid mice climb to their tails. For the FST, the experimental mice were placed in a clear glass cylinder (height: 25 cm; diameter: 10 cm) filled to 10 cm with water at 25 ± 1°C for 6 min and were considered immobile when they make only necessary movements to keep their heads above the water. The duration of immobility during the last 4 min of suspension or forced swimming was recorded by a video (Zhenghua, Anhui, China). In the FST, the water in the glass cylinder was replaced after each trial. Behavioral assays were carried out between 9:00 and 11:00 a.m. to avoid circadian variation.

### Intracerebroventricular Infusion of LY294002

The intracerebroventricular infusion of LY294002 was conducted according to our previous studies (Huang et al., 2018; Wang et al., 2018). Briefly, the mice were anaesthetized with pentobarbital sodium and secured onto a stereotactic head frame (Quintessential Stereotaxic Injector, STELING Corporation, DALE, IL, United States). The cannulas were implanted into the left lateral brain ventricle (−0.2 mm anterior and 1.0 mm lateral relative to bregma and 2.3 mm below the surface of the skull) (Kleinridders et al., 2009) and connected to an osmotic minipump (Alzet model 2002 for chronic injections and Alzet model 1003D for acute injections, Alza Corporation, Cupertino, CA, United States). Minipumps were filled with 5 μg/ml of LY294002 (dissolved in 3% DMSO) or vehicle in sterile artificial cerebrospinal fluid (ACSF, 0.1 μg per day) and were implanted subcutaneously in the interscapular region (injection rate 0.5 μL/h). The dose of LY294002 was in the range of that reported in previous studies (Quesada et al., 2008; Fortress et al., 2013).

### Statistical Analysis

Statistical analyses were performed using Graphpad Prism 8 (Graphpad Software, Inc., La Jolla, CA, United States). Differences between mean values were evaluated using a one-way analysis of variance (ANOVA) or two-way ANOVA, as appropriate, followed by a post-hoc Bonferroni test to assess isolated comparisons. When any two factors in the experiment did not interact (e.g., *p* > 0.05) in the ANOVA, we used a t-test that has been described by Wei et al. (2012) to make further comparisons between different factors (Wei et al., 2012). Data are expressed as mean ± standard error of mean (SEM). Differences were considered significant at *p* < 0.05.

## Results

### KRIBB11 Reversibly Induces Microglial Process Elongation at Conditions *in vitro* and *in vivo*

Based on our preliminary observations, we first investigated the dose-dependent effect of KRIBB11 on microglial process. 5 h of KRIBB11 incubation at the dose of 1 and 3 μM but not 0.5 μM was found to induce remarkable elongations of the processes in the primary cultured microglia (F_3,116_ = 8.15, *p* < 0.001, Figures 1A,B) with no significant toxic effects observed in MTT assays (F_3,28_ = 0.47, *p* = 0.71, Figure 1C). The time-dependent analysis showed that it was the 3 and 5 h but not 1 h of KRIBB11 incubation (3 μM) induced remarkable elongations of the processes in the primary cultured microglia (F_3,116_ = 12.49, *p* < 0.001, Figures 1D,E) with no significant toxic effects observed in MTT assays (F_3,28_ = 1.43, *p* = 0.25, Figure 1F). Further analysis showed that the length of the processes in the primary cultured microglia incubated with 5 h of KRIBB11 (3 μM) returned to the basal levels 24 h after KRIBB11 washout (F_2,87_ = 34.57, *p* < 0.001, Figures 2B,C). The pro-elongation effect of KRIBB11 on microglial process was also observed at conditions *in vivo*: 5 days of KRIBB11 injection at the dose of 5 mg/kg induced remarkable elongations of the processes in the microglia in the dorsolateral prefrontal cortex in mice (Figures 2D,E). Moreover, we found that the length of the processes in microglia in the dorsolateral prefrontal cortex in mice injected with 5 days of KRIBB11 (once daily, 5 mg/kg) returned to the basal levels 5 days after the discontinuation of KRIBB11 treatment (F_2,87_ = 20.84, *p* < 0.001, Figures 2D,E). These results demonstrated that the pro-elongation effect of KRIBB11 on microglial process is reversible.

### Pro-Elongation Effect of KRIBB11 on Microglial Process at Inflammatory Conditions

**FIGURE 3:**
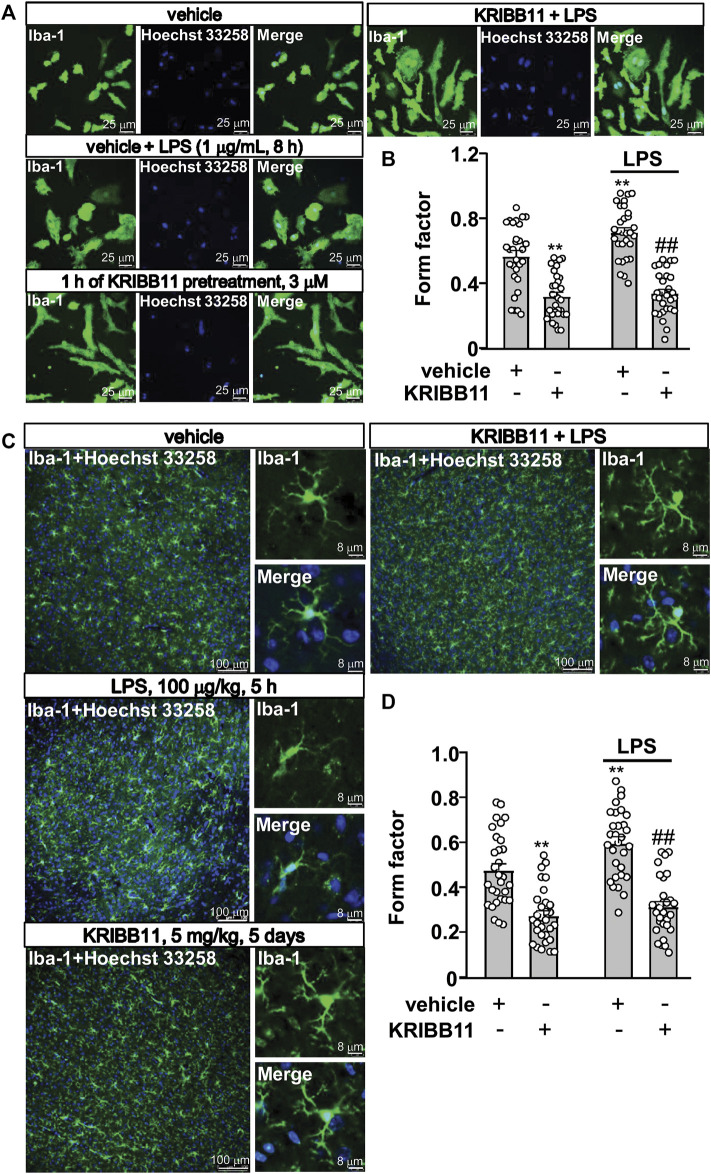
Pro-elongation effects of KRIBB11 on microglial process in inflammatory conditions. **(A)** Representative images showed that KRIBB11 pretreatment (3 μM, 1 h) prevented LPS (1 μg/ml, 8 h)-induced retractions of primary cultured microglial process. **(B)** Quantitative analysis showed the changes in the form factor in primary cultured microglia treated with or without LPS and/or KRIBB11 (**p < 0.01 vs. vehicle, ##*p* < 0.01 vs. vehicle + LPS). **(C)** Representative images showed that KRIBB11 pretreatment (5 mg/kg, 5 days) prevented LPS (100 μg/kg, 5 h)-induced retractions of microglial process in the dorsolateral prefrontal cortex. **(D)** Quantitative analysis showed the changes in the form factor in microglial processes in the dorsolateral prefrontal cortex in mice treated with or without LPS and/or KRIBB11 (**p < 0.01 vs. vehicle, ##*p* < 0.01 vs. vehicle + LPS). For cell shape investigation at conditions *in vitro* and *in vivo*, 30 cells per condition were analyzed in three independent experiments. Scale bars for *in vitro* analysis is 25 μm, and scale bars for *in vivo* analysis in the low and high magnification images are 100 and 8 μm, respectively. Data are shown as mean ± SEM.

In pathological conditions, the microglia usually display amoeboid morphologies with retracted processes. Thus, we examined whether KRIBB11 can prevent LPS-induced changes in microglial morphology. To this end, the primary cultured microglia were pre-incubated with KRIBB11 for 1 h (3 μM), and then stimulated with 1 μg/ml of LPS for 8 h. A two-way ANOVA for the length of the processes in the primary cultured microglia showed significant effects for LPS treatment (F_1,116_ = 8.35, *p* < 0.01), KRIBB11/vehicle pretreatment (F_1,116_ = 116.50, *p* < 0.001), and the LPS × KRIBB11/vehicle interaction (F_1,116_ = 4.89, *p* < 0.05) (Figures 3A,B). Post hoc analysis revealed that KRIBB11 pre-incubation (3 μM, 1 h) prevented the LPS (1 μg/ml, 8 h)-induced shortenings of the processes in the primary cultured microglia (Figures 3A,B).

Then, we examined the above mentioned phenomenon can occur at conditions *in vivo*. The mice were pre-treated with KRIBB11 (5 mg/kg) for 5 days, after which the mice accepted a single dose injection of LPS (100 μg/kg). 5 h after LPS injection, the mice were sacrificed for brain collections for immunofluorescence. The two-way ANOVA for the length of the processes in the microglia in the dorsolateral prefrontal cortex showed significant effects for LPS treatment (F_1,116_ = 9.74, *p* < 0.01), KRIBB11/vehicle pretreatment (F_1,116_ = 89.02, *p* < 0.001) but not the LPS × KRIBB11/vehicle interaction (F_1,116_ = 2.49, *p* = 0.12) (Figures 3A,B). Post hoc analysis revealed that KRIBB11 pretreatment (5 mg/kg, 5 days) prevented the LPS (100 μg/kg, 5 h)-induced shortenings of the processes in the microglia in the dorsolateral prefrontal cortex (Figures 3C,D). These results demonstrated that the KRIBB11 can render the microglia to resist against process retractions induced by pro-inflammatory stimuli.

### Preventive Effect of KRIBB11 on Lipopolysaccharide-Induced Inflammatory Responses in Primary Cultured Microglia

**FIGURE 4:**
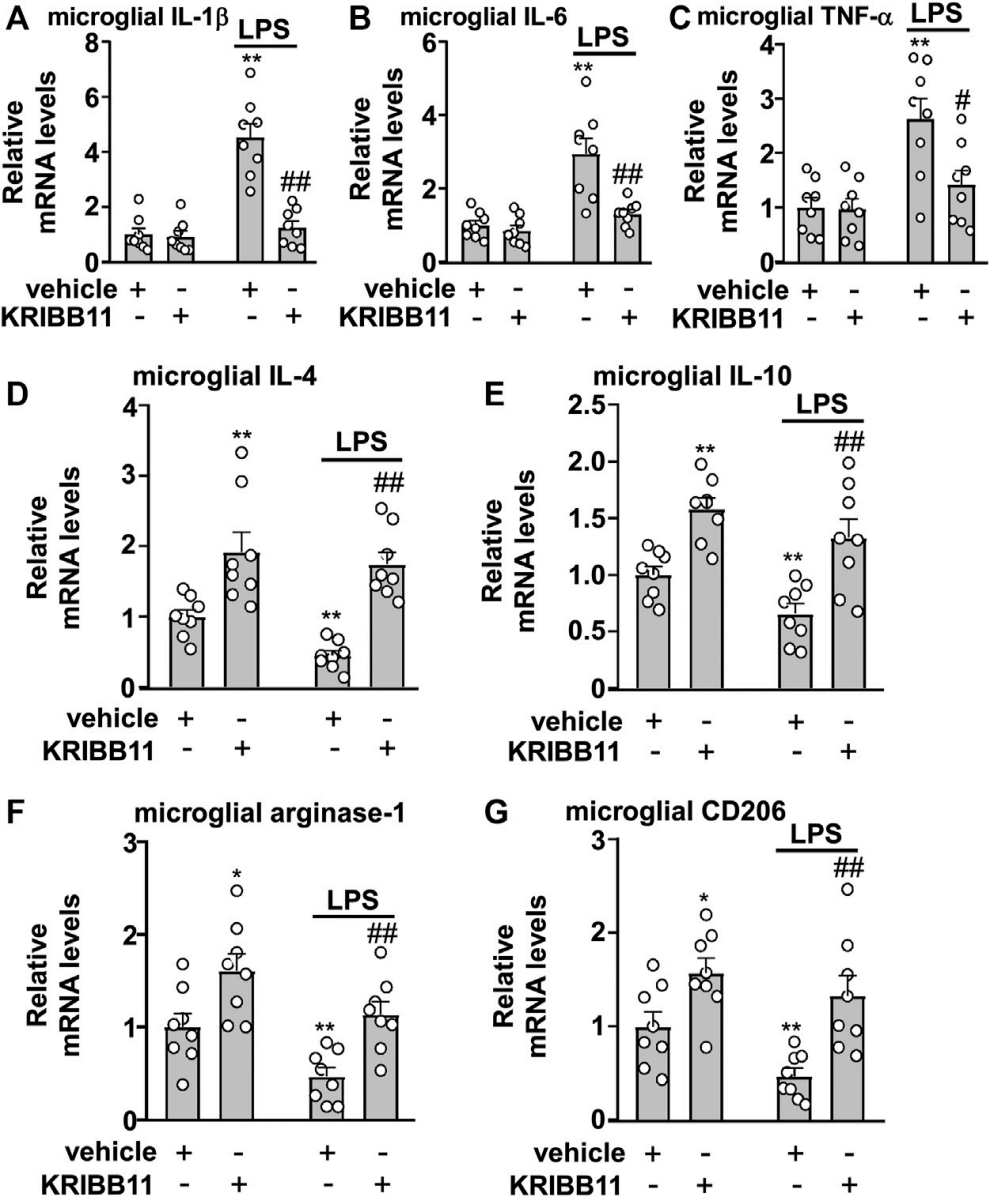
Effects of KRIBB11 on LPS-induced inflammatory responses in primary cultured microglia. **(A‒C)** Quantitative analysis showed the preventive effect of KRIBB11 pretreatment (3 μM, 1 h) on LPS (1 μg/ml, 8 h)-induced increases in the expression levels of IL-1β **(A)**, IL-6 **(B)**, and TNF-α **(C)** mRNA in primary cultured microglia (*n* = 8, **p < 0.01 vs. vehicle, #*p* < 0.05 or ##*p* < 0.01 vs. vehicle + LPS). **(D‒G)** Quantitative analysis showed the preventive effect of KRIBB11 pretreatment (3 μM, 1 h) on the expression levels of IL-4 **(D)**, IL-10 **(E)**, arginase-1 **(F)**, and CD206 **(G)** mRNA in primary cultured microglia treated with or without LPS (1 μg/ml, 8 h; *n* = 8, *p < 0.05 or **p < 0.01 vs. vehicle, ##*p* < 0.01 vs. vehicle + LPS). Data are shown as mean ± SEM.

Considering that the elongation of microglial process is associated with anti-neuroinflammation, we assumed that KRIBB11 incubation may prevent the progression of inflammatory responses in microglia. To examine this hypothesis, the primary cultured microglia were pre-incubated with KRIBB11 for 1 h (3 μM), and then stimulated with 1 μg/ml of LPS for 8 h. As shown in Figures 4A–C, a two-way ANOVA for the expression levels of IL-1β, IL-6, and TNF-α mRNA in the primary cultured microglia incubated with or without LPS and/or KRIBB11 showed significant effects for LPS treatment (IL-1β: F_1,28_ = 38.64, *p* < 0.001; IL-6: F_1,28_ = 25.18, *p* < 0.001; TNF-α: F_1,28_ = 15.92, *p* < 0.001), KRIBB11/vehicle pretreatment (IL-1β: F_1,28_ = 29.31, *p* < 0.001; IL-6: F_1,28_ = 13.97, *p* < 0.001; TNF-α: F_1,28_ = 5.76, *p* < 0.05), and the LPS × KRIBB11/vehicle interaction (IL-1β: F_1,28_ = 26.29, *p* < 0.001; IL-6: F_1,28_ = 9.79, *p* < 0.01; TNF-α: F_1,28_ = 5.16, *p* < 0.05). Post hoc analysis revealed that KRIBB11 pretreatment (3 μM) prevented the LPS (1 μg/ml, 8 h)-induced increases in the expression levels of IL-1β (Figure 4A), IL-6 (Figure 4B), and TNF-α (Figure 4C) mRNA in the primary cultured microglia.

In Figures 4D‒G, the two-way ANOVA for the expression levels of IL-4, IL-10, arginase-1, and CD206 mRNA in the primary cultured microglia incubated with or without LPS and/or KRIBB11 showed significant effects for LPS treatment (IL-4: F_1,28_ = 4.41, *p* < 0.05; IL-10: F_1,28_ = 7.03, *p* < 0.05; arginase-1: F_1,28_ = 12.34, *p* < 0.01; CD206: F_1,28_ = 5.95, *p* < 0.05) and KRIBB11/vehicle pretreatment (IL-4: F_1,28_ = 40.53, *p* < 0.001; IL-10: F_1,28_ = 31.15, *p* < 0.001; arginase-1: F_1,28_ = 19.83, *p* < 0.001; CD206: F_1,28_ = 20.32, *p* < 0.001), but not for the LPS × KRIBB11/vehicle interaction (IL-4: F_1,28_ = 1.16, *p* = 0.29; IL-10: F_1,28_ = 0.16, *p* = 0.69; arginase-1: F_1,28_ = 0.04, *p* = 0.84; CD206: F_1,28_ = 0.82, *p* = 0.37). Post hoc analysis revealed that KRIBB11 pretreatment (3 μM) prevented the LPS-induced decreases in the expression levels of IL-4 (Figure 4D), IL-10 (Figure 4E), arginase-1 (Figure 4F), and CD206 (Figure 4G) mRNA in the primary cultured microglia. These results demonstrated that the KRIBB11 possesses an ability to prevent the pro-inflammatory responses in microglia.

### Preventive Effect of KRIBB11 on LPS-Induced Neuroinflammatory Responses and Depression-like Behaviors in Mice

**FIGURE 5:**
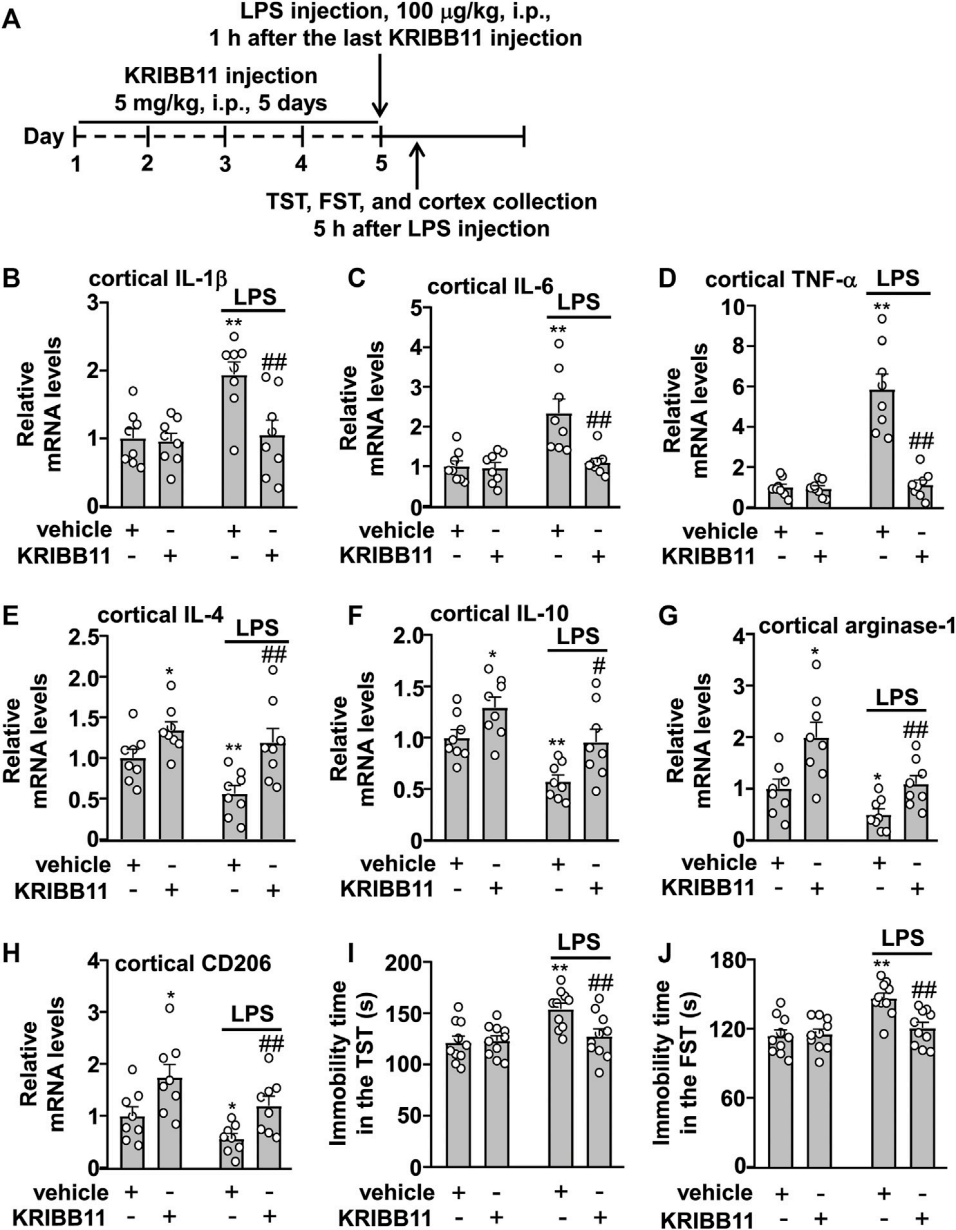
Effects of KRIBB11 on LPS-induced inflammatory responses in the dorsolateral prefrontal cortex and depression-like behaviors in mice. **(A)** Time-line showing the investigation of the effects of KRIBB11 on LPS-induced inflammatory responses in the dorsolateral prefrontal cortex and depression-like behaviors. **(B‒D)** Quantitative analysis showed the preventive effect of KRIBB11 pretreatment (5 mg/kg, 5 days) on LPS (100 μg/kg, 5 h)-induced increases in the expression levels of IL-1β **(B)**, IL-6 **(C)**, and TNF-α **(D)** mRNA in the dorsolateral prefrontal cortex (*n* = 8, **p < 0.01 vs. vehicle, ##*p* < 0.01 vs. vehicle + LPS). **(E‒H)** Quantitative analysis showed the effect of KRIBB11 pretreatment (5 mg/kg, 5 days) on the expression levels of IL-4 **(E)**, IL-10 **(F)**, arginase-1 **(G)**, and CD206 **(H)** mRNA in the dorsolateral prefrontal cortex in mice treated with or without LPS (100 μg/kg, 5 h; *n* = 8, *p < 0.05 or **p < 0.01 vs. vehicle, #*p* < 0.05 or ##*p* < 0.01 vs. vehicle + LPS). **(I,J)** Quantitative analysis showed the preventive effect of KRIBB11 pretreatment (5 mg/kg, 5 days) on LPS (100 μg/kg, 5 h)-induced increases in the immobility time in the TST **(I)** and FST **(J)** in mice (*n* = 10, **p < 0.01 vs. vehicle, ##*p* < 0.01 vs. vehicle + LPS). Data are shown as mean ± SEM.

Next, we examined whether KRIBB11 pretreatment can prevent the neuroinflammatory response and neuroinflammation-associated behavioral deficits. To this end, the mice were firstly pre-treated with KRIBB11 (5 mg/kg) for 5 days and then accepted a single dose of LPS injection (100 μg/kg). 5 h after LPS injection, the mice were sacrificed for cortex collections for mRNA detection (Figure 5A). A two-way ANOVA for the expression levels of IL-1β, IL-6, and TNF-α mRNA in the dorsolateral prefrontal cortex in mice treated with or without LPS and/or KRIBB11 showed significant effects for LPS treatment (IL-1β: F_1,28_ = 9.28, *p* < 0.01; IL-6: F_1,28_ = 12.27, *p* < 0.01; TNF-α: F_1,28_ = 37.57, *p* < 0.001), KRIBB11/vehicle pretreatment (IL-1β: F_1,28_ = 7.53, *p* < 0.05; IL-6: F_1,28_ = 9.32, *p* < 0.01; TNF-α: F_1,28_ = 33.97, *p* < 0.001), and the LPS × KRIBB11/vehicle interaction (IL-1β: F_1,28_ = 6.19, *p* < 0.05; IL-6: F_1,28_ = 8.25, *p* < 0.01; TNF-α: F_1,28_ = 32.10, *p* < 0.001) (Figures 5B‒D). Post hoc analysis revealed that KRIBB11 pretreatment (5 mg/kg) could prevent the LPS (100 μg/kg)-induced increases in the expression levels of IL-1β (Figure 5B), IL-6 (Figure 5C), and TNF-α (Figure 5D) mRNA in the dorsolateral prefrontal cortex in mice.

The two-way ANOVA for the expression levels of IL-4, IL-10, arginase-1, and CD206 mRNA in the dorsolateral prefrontal cortex in mice treated with or without LPS and/or KRIBB11 showed significant effects for LPS treatment (IL-4: F_1,28_ = 5.77, *p* < 0.05; IL-10: F_1,28_ = 15.98, *p* < 0.001; arginase-1: F_1,28_ = 12.40, *p* < 0.01; CD206: F_1,28_ = 7.09, *p* < 0.05) and KRIBB11/vehicle pretreatment (IL-4: F_1,28_ = 15.53, *p* < 0.001; IL-10: F_1,28_ = 12.44, *p* < 0.01; arginase-1: F_1,28_ = 16.05, *p* < 0.001; CD206: F_1,28_ = 13.66, *p* < 0.001) but not for the LPS × KRIBB11/vehicle interaction (IL-4: F_1,28_ = 1.33, *p* = 0.26; IL-10: F_1,28_ = 0.21, *p* = 0.65; arginase-1: F_1,28_ = 1.02, *p* = 0.32; CD206: F_1,28_ = 0.09, *p* = 0.77) (Figures 5E‒H). Post hoc analysis revealed that KRIBB11 pretreatment (5 mg/kg) prevented the LPS (100 μg/kg)-induced decreases in the expression levels of IL-4 (Figure 5E), IL-10 (Figure 5F), arginase-1 (Figure 5G), and CD206 (Figure 5H) mRNA in the dorsolateral prefrontal cortex in mice.

As high levels of neuroinflammatory responses can induce behavioral deficits, we investigated whether KRIBB11 pretreatment prevents LPS-induced depression-like behaviors. A two-way ANOVA for the immobility time in the TST and FST in mice treated with or without LPS and/or KRIBB11 showed significant effects for LPS treatment (TST: F_1,36_ = 9.46, *p* < 0.01; FST: F_1,36_ = 15.64, *p* < 0.001), KRIBB11/vehicle pretreatment (TST: F_1,36_ = 4.14, *p* < 0.05; FST: F_1,36_ = 6.51, *p* < 0.05), and the LPS × KRIBB11/vehicle interaction (TST: F_1,36_ = 5.50, *p* < 0.05; FST: F_1,36_ = 8.19, *p* < 0.01) (Figures 5I,J). Post hoc analysis revealed that KRIBB11 pretreatment (5 mg/kg) prevented the LPS (100 μg/kg)-induced increases in the immobility time in the TST (Figure 5I) and FST (Figure 5J) in mice. Taken together, these results demonstrated that KRIBB11 pretreatment can prevent the LPS-induced neuroinflammatory response and depression-like behaviors in mice.

### Activating Protein Kinase B Activation Mediates the Pro-Elongation Effect of KRIBB11 on Primary Cultured Microglial Processes

**FIGURE 6:**
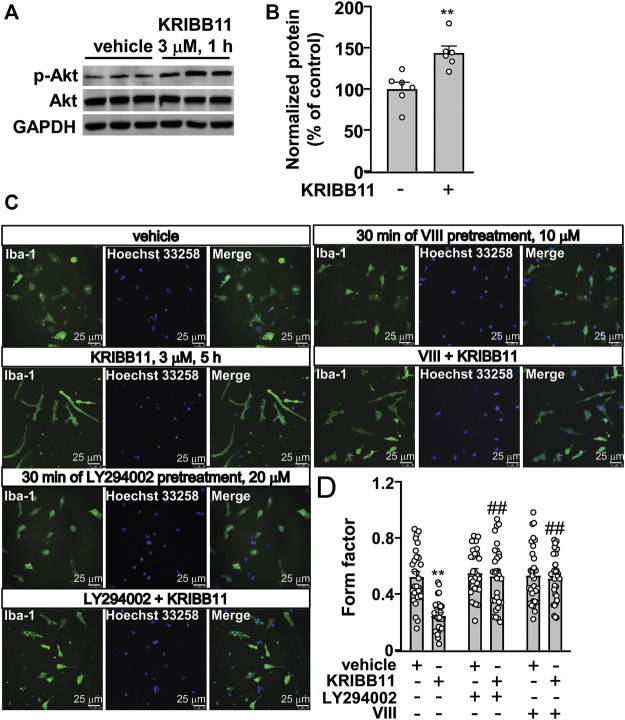
Akt activation mediates the pro-elongation effect of KRIBB11 on primary cultured microglial process. **(A,B)** Representative images **(A)** and quantitative analysis **(B)** showed that KRIBB11 incubation (3 μM, 30 min) induced a significant increase in the phosphorylation levels of Akt in primary cultured microglia (*n* = 6, **p < 0.01 vs. vehicle). **(C,D)** Representative images **(C)** and quantitative analysis **(D)** showed that inhibition of Akt activity by LY294002 (20 μM, 30 min) or VIII (10 μM, 30 min) pre-incubation prevented the pro-elongation effect of KRIBB11 (3 μM, 5 h) on primary cultured microglial process (**p < 0.01 vs. vehicle, ##*p* < 0.01 vs. vehicle + KRIBB11). For cell shape investigation, 30 cells per condition were analyzed in three independent experiments. Scale bars: 25 μm. Data are shown as mean ± SEM.

Our previous studies had reported that Akt activation is required for the pro-elongation effect of different molecules, such as butyrate and β-3-hydrobutyrate, on microglial process (Huang et al., 2018; Wang et al., 2018). Here, we hypothesized that the activation of Akt may mediate the pro-elongation effect of KRIBB11 on microglial process. We first checked influence of KRIBB11 on Akt phosphorylation in primary cultured microglia. Results showed that KRIBB11 treatment (3 μM, 1 h) induced a significant increase in the phosphorylation levels of Akt in the primary cultured microglia (t_10_ = 3.85, *p* < 0.01) (Figures 6A,B). Then, we used the Akt inhibitor, LY294002 and VIII, to investigate the obligatory role of Akt in the pro-elongation effect of KRIBB11 on microglial process. In this experiment, a two-way ANOVA showed significant effects for KRIBB11 incubation (F_1,174_ = 16.03, *p* < 0.001), Akt inhibitor pretreatment (F_2,174_ = 13.24, *p* < 0.001), and the KRIBB11 × Akt inhibitor interaction (F_2,174_ = 9.97, *p* < 0.001) (Figures 6C,D). Post hoc analysis revealed that inhibition of Akt by LY294002 (20 μM, Figures 6C,D) or VIII (10 μM, Figures 6C,D) pretreatment blocked the pro-elongation effect of KRIBB11 on primary cultured microglial process.

### Activating Protein Kinase B Inhibition Abrogates the Regulatory Effect of KRIBB11 on Inflammatory Responses in Primary Cultured Microglia

**FIGURE 7:**
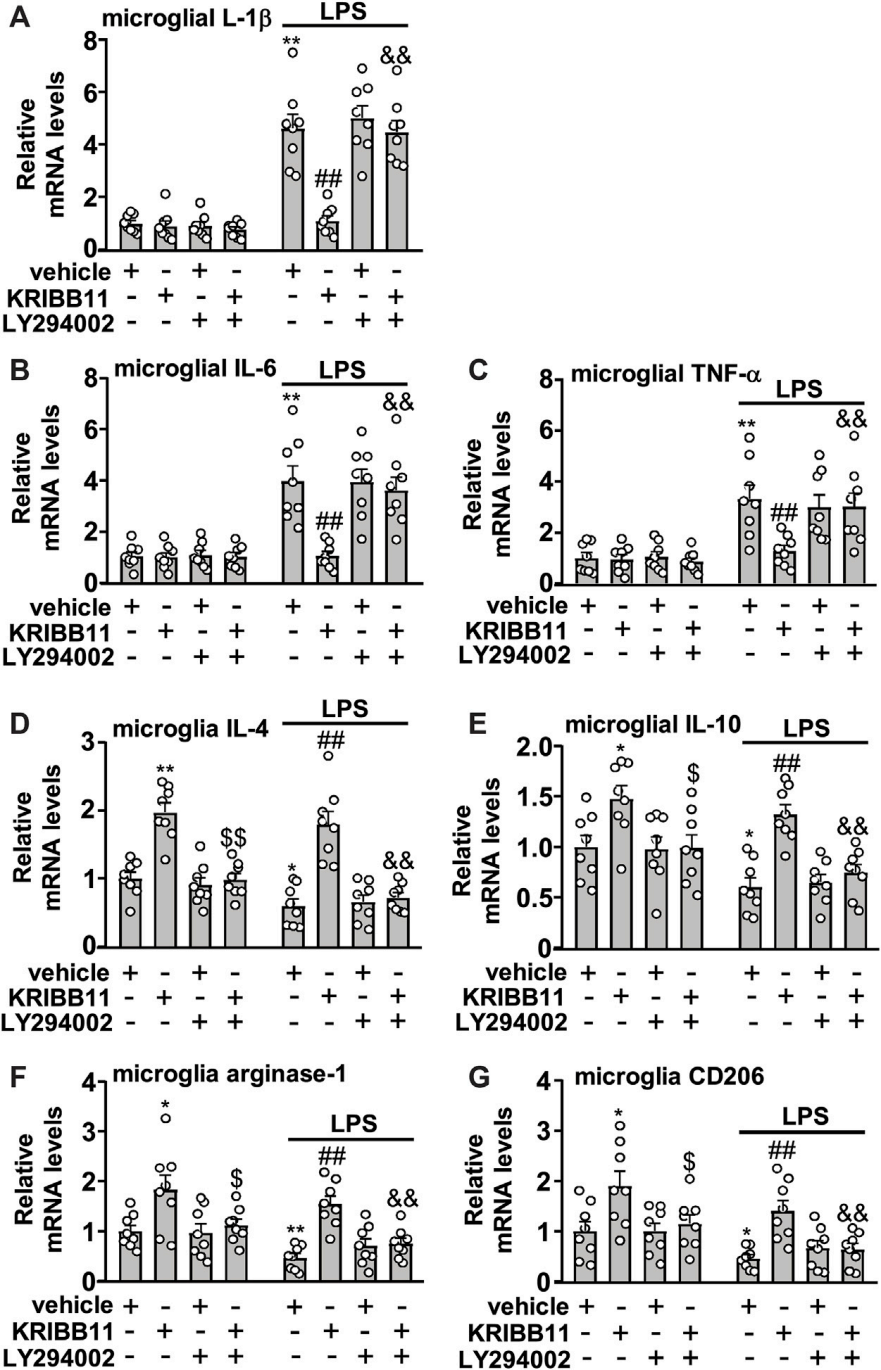
Akt inhibition abrogates the preventive effect of KRIBB11 on LPS-induced inflammatory responses in primary cultured microglia. **(A‒C)** Quantitative analysis showed that LY294002 pretreatment (20 μM, 30 min) abrogated the preventive effect of KRIBB11 on LPS-induced increases in the expression levels of IL-1β **(A)**, IL-6 **(B)**, and TNF-α **(C)** mRNA in primary cultured microglia (*n* = 8, **p < 0.01 vs. vehicle, ##*p* < 0.01 vs. vehicle + LPS; &&*p* < 0.01 vs. KRIBB11 + LPS). **(D‒G)** Quantitative analysis showed the abrogation effect of LY294002 pretreatment (20 μM, 30 min) on KRIBB11-induced increases in the expression levels of IL-4 **(D)**, IL-10 **(E)**, arginase-1 **(F)**, and CD206 **(G)** mRNA in primary cultured microglia treated with or without LPS (*n* = 8, *p < 0.05 or **p < 0.01 vs. vehicle, $*p* < 0.05 or $$*p* < 0.01 vs. KRIBB11, ##*p* < 0.01 vs. vehicle + LPS; &&*p* < 0.01 vs. KRIBB11 + LPS). Data are shown as mean ± SEM.

We also investigated whether the inhibition of microglial process elongation could influence the regulatory effect of KRIBB11 on inflammatory responses. In experiments involving the measurement of the expression levels of IL-1β, IL-6, and TNF-α mRNA in primary cultured microglia incubated with or without KRIBB11, LY294002, and/or LPS, a two-way ANOVA showed significant effects for LPS treatment (IL-1β: F_1, 56_ = 164.70, *p* < 0.001; IL-6: F_1, 56_ = 73.50, *p* < 0.001; TNF-α: F_1, 56_ = 46.94, *p* < 0.001), KRIBB11/LY294002/vehicle incubation (IL-1β: F_3, 56_ = 16.10, *p* < 0.001; IL-6: F_3, 56_ = 8.47, *p* < 0.001; TNF-α: F_3, 56_ = 3.66, *p* < 0.05), and the LPS × KRIBB11/LY294002/vehicle interaction (IL-1β: F_3, 56_ = 16.07, *p* < 0.001; IL-6: F_3, 56_ = 7.93, *p* < 0.001; TNF-α: F_3, 56_ = 3.48, *p* < 0.0) (Figures 7A,B). Post hoc analysis revealed that pre-incubation of the primary cultured microglia with LY294002 (20 μM) abrogated the reversal effect of KRIBB11 (3 μM) on LPS (1 μg/ml)-induced increases in the expression levels of IL-1β (Figure 7A), IL-6 (Figure 7B), and TNF-α (Figure 7C) mRNA.

In the experiments involving the measurement of anti-inflammatory mediators, the two-way ANOVA for the expression levels of IL-4, IL-10, arginase-1, and CD206 mRNA in primary cultured microglia incubated with or without KRIBB11, LY294002, and/or LPS showed significant effects for LPS treatment (IL-4: F_1,56_ = 10.96, *p* < 0.01; IL-10: F_1,56_ = 14.03, *p* < 0.001; arginase-1: F_1,56_ = 10.09, *p* < 0.01; CD206: F_1,56_ = 13.80, *p* < 0.001) and KRIBB11/LY294002/vehicle incubation (IL-4: F_3,56_ = 42.04, *p* < 0.05; IL-10: F_3,56_ = 14.46, *p* < 0.001; arginase-1: F_3,56_ = 14.40, *p* < 0.001; CD206: F_3,56_ = 10.99, *p* < 0.001) but not for the LPS × KRIBB11/LY294002/vehicle interaction (IL-4: F_3,56_ = 0.32, *p* = 0.81; IL-10: F_3,56_ = 0.48, *p* = 0.76; arginase-1: F_3,56_ = 0.29, *p* = 0.83; CD206: F_3,56_ = 0.12, *p* = 0.95) (Figures 7D‒G). Post hoc analysis revealed that compared with the KRIBB11/LPS-treated microglia, the primary cultured microglia co-treated with LY294002, KRIBB11, and LPS exhibited significant decreases in the expression levels of IL-4 (Figure 7D), IL-10 (Figure 7E), arginase-1 (Figure 7F), and CD206 (Figure 7G) mRNA.

### Activating Protein Kinase B Inhibition Abrogates the Pro-elongation Effect of KRIBB11 on Microglial Process in the Dorsolateral Prefrontal Cortex

**FIGURE 8:**
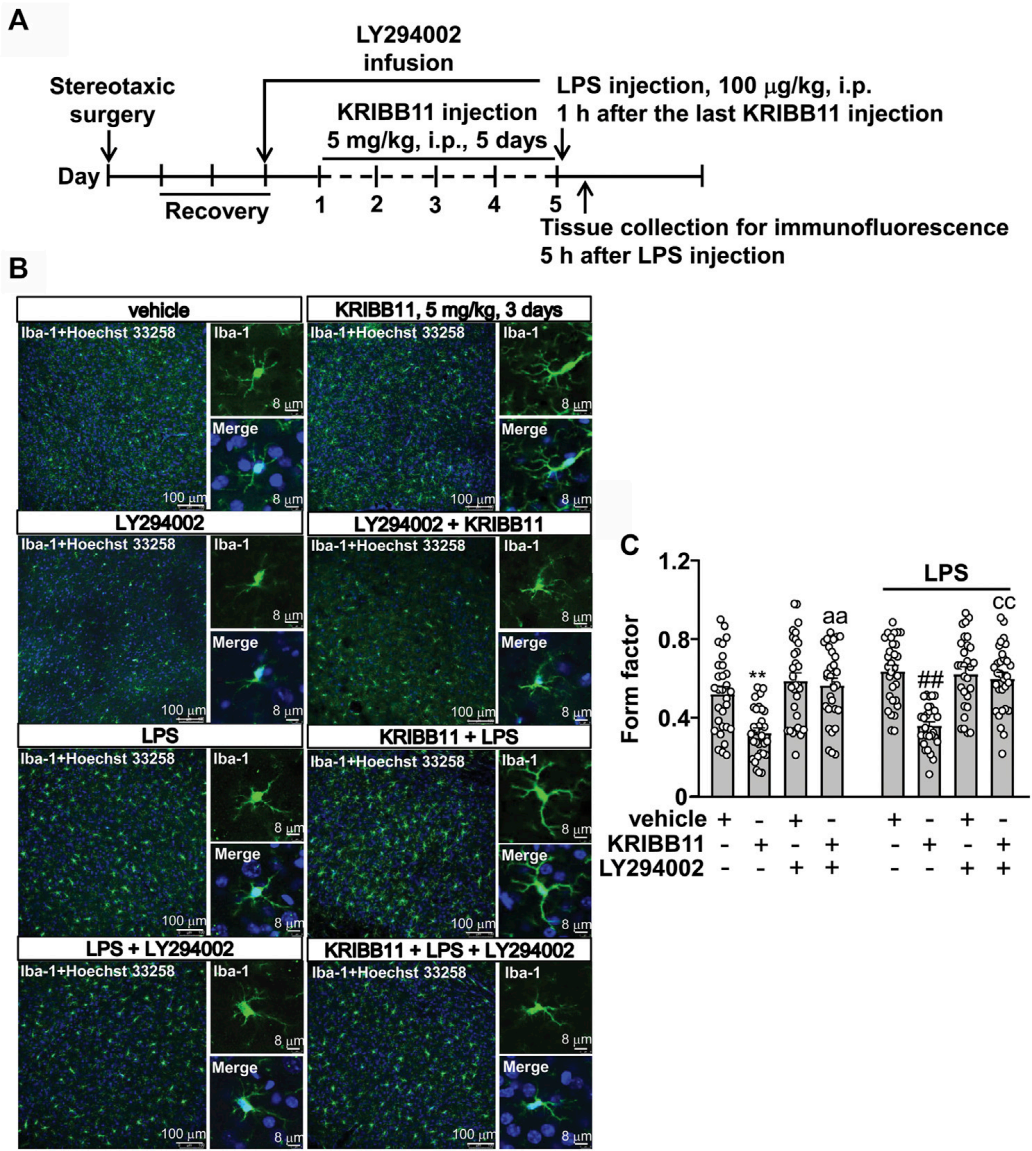
LY294002 infusion abrogates the pro-elongation effect of KRIBB11 on microglial process in the dorsolateral prefrontal cortex in mice treated with or without LPS. **(A)** Time-line showing the investigation of the effects of intracerebroventricularly LY294002 infusion on the pro-elongation effect of KRIBB11 on microglial process in the dorsolateral prefrontal cortex in mice treated with or without LPS. **(B,C)** Representative images **(B)** and quantitative analysis **(C)** showed that intracerebroventricularly LY294002 infusion abrogated the pro-elongation effect of KRIBB11 on microglial process in the dorsolateral prefrontal cortex in mice treated with or without LPS (**p < 0.01 vs. vehicle, ##*p* < 0.01 vs. vehicle + LPS, aa*p* < 0.01 vs. KRIBB11, cc*p* < 0.01 vs. KRIBB11 + LPS). For cell shape investigation, 30 cells per condition were analyzed in three independent experiments. Scale bars in the low and high magnification images are 100 and 8 μm, respectively. Data are shown as mean ± SEM.

To investigate whether Akt activation is involved in the pro-elongation effect of KRIBB11 on microglia process at conditions *in vivo*, we used the intracerebroventricular infusion method to inhibit the activity of Akt in the brain (Figure 8A). As shown in Figures 8B,C, a two-way ANOVA showed significant effects for LPS injection (F_1,232_ = 6.18, *p* < 0.05), KRIBB11/LY294002/vehicle treatment (F_3,232_ = 31.11, *p* < 0.001), but not for the LPS × KRIBB11/LY294002/vehicle interaction (F_3,232_ = 0.81, *p* = 0.49). Post hoc analysis revealed that the intracerebroventricular LY294002 infusion prevented the KRIBB11 (5 mg/kg)-induced elongations of the processes in the microglia in the dorsolateral prefrontal cortex in both stress-naïve and LPS-stimulated mice (Figures 8B,C).

### Activating Protein Kinase B Inhibition Abrogates the Preventive Effect of KRIBB11 on LPS-Induced Neuroinflammatory Responses in the Brain and Depression-Like Behaviors in Mice

**FIGURE 9:**
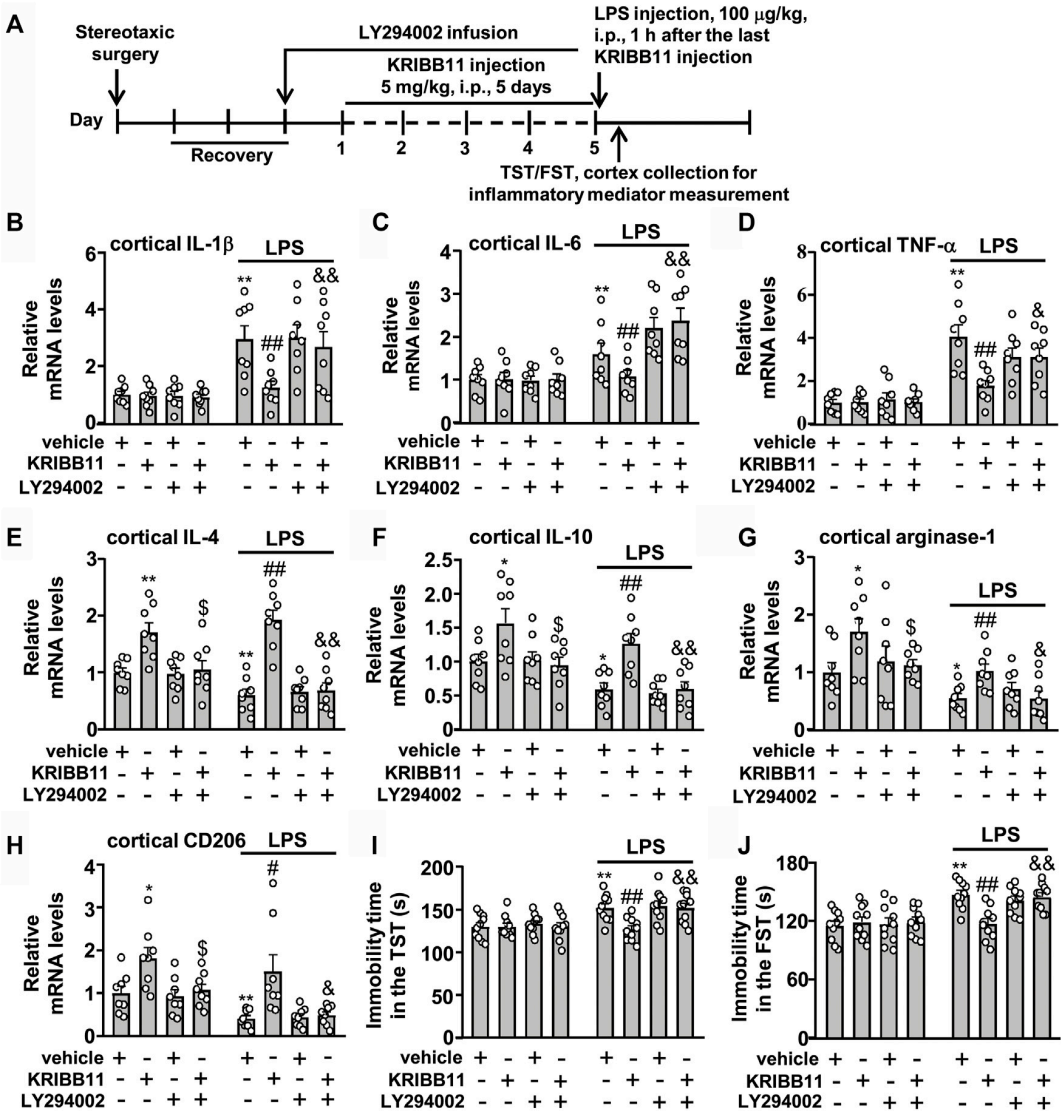
LY294002 infusion abrogates the preventive effect of KRIBB11 on LPS-induced neuroinflammatory responses and depression-like behaviors in mice. **(A)** Time-line showing the investigation of the effect of intracerebroventricular LY294002 infusion on the preventive effect of KRIBB11 on LPS-induced neuroinflammatory responses and depression-like behaviors. **(B‒D)** Quantitative analysis showed that intracerebroventricularly LY294002 infusion abrogated the preventive effect of KRIBB11 on LPS-induced increases in the expression levels of IL-1β **(B)**, IL-6 **(C)**, and TNF-α **(D)** mRNA in the dorsolateral prefrontal cortex in mice (*n* = 8, **p < 0.01 vs. vehicle, ##*p* < 0.01 vs. vehicle + LPS, &*p* < 0.05 or &&*p* < 0.01 vs. KRIBB11 + LPS). **(E‒H)** Quantitative analysis showed the abrogation effect of intracerebroventricular LY294002 infusion on KRIBB11-induced increases in the expression levels of IL-4 **(E)**, IL-10 **(F)**, arginase-1 **(G)**, and CD206 **(H)** mRNA in the dorsolateral prefrontal cortex in mice treated with or without LPS (*n* = 8, *p < 0.05 or **p < 0.01 vs. vehicle; $*p* < 0.05 vs. KRIBB11; #*p* < 0.05 or ##*p* < 0.01 vs. vehicle + LPS; &*p* < 0.05 or &&*p* < 0.01 vs. KRIBB11 + LPS). **(I,J)** Quantitative analysis showed that intracerebroventricular LY294002 infusion abrogated the preventive effect of KRIBB11 on LPS-induced increases in the immobility tine in the TST **(I)** and FST **(J)** in mice (*n* = 10, **p < 0.01 vs. vehicle, ##*p* < 0.01 vs. vehicle + LPS, &&*p* < 0.01 vs. KRIBB11 + LPS). Data are shown as mean ± SEM.

Finally, we investigated whether Akt inhibition could abrogate the preventive effect of KRIBB11 on LPS-induced neuroinflammatory responses and depression-like behaviors. We first evaluated the effect of LY294002 on the preventive effect of KRIBB11 on LPS-induced neuroinflammatory responses in the dorsolateral prefrontal cortex (Figure 9A). A two-way ANOVA for the expression levels of IL-1β, IL-6, and TNF-α mRNA showed significant effects for LPS injection (IL-1β: F_1,56_ = 44.74, *p* < 0.001; IL-6: F_1,56_ = 37.17, *p* < 0.001; TNF-α: F_1,56_ = 70.32, *p* < 0.001), KRIBB11/LY294002/vehicle treatment (IL-1β: F_3,56_ = 3.44, *p* < 0.05; IL-6: F_3,56_ = 4.74, *p* < 0.01; TNF-α: F_3,56_ = 4.01, *p* < 0.05), and the LPS × KRIBB11/LY294002/vehicle interaction (IL-1β: F_3,56_ = 3.38, *p* < 0.05; IL-6: F_3,56_ = 5.07, *p* < 0.01; TNF-α: F_3,56_ = 4.12, *p* < 0.05) (Figures 9B‒D). Post hoc analysis revealed that the intracerebroventricular LY294002 infusion abrogated the preventive effect of KRIBB11 on LPS-induced increases in the expression levels of IL-1β (Figure 9B), IL-6 (Figure 9C), and TNF-α (Figure 9D) mRNA in the dorsolateral prefrontal cortex.

For the expression levels of IL-4, IL-10, arginase-1, and CD206 mRNA, the two-way ANOVA showed significant effects for LPS injection (IL-4: F_1,56_ = 5.53, *p* < 0.05; IL-10: F_1,56_ = 18.14, *p* < 0.001; arginase-1: F_1,56_ = 22.94, *p* < 0.001; CD206: F_1,56_ = 12.88, *p* < 0.001), KRIBB11/LY294002/vehicle treatment (IL-4: F_3,56_ = 29.36, *p* < 0.001; IL-10: F_3,56_ = 12.14, *p* < 0.001; arginase-1: F_3,56_ = 5.38, *p* < 0.01; CD206: F_1,56_ = 11.68, *p* < 0.001), but not for the LPS × KRIBB11/LY294002/vehicle interaction (IL-4: F_3,56_ = 2.55, *p* = 0.07; IL-10: F_3,56_ = 0.20, *p* = 0.90; arginase-1: F_1,56_ = 0.21, *p* = 0.89; CD206: F_1,56_ = 0.24, *p* = 0.87) (Figures 9E‒H). Post hoc analysis revealed that the intracerebroventricular LY294002 infusion abrogated the KRIBB11-induced increases in the expression levels of IL-4 (Figure 9E), IL-10 (Figure 9F), arginase-1 (Figure 9G), and CD206 (Figure 9H) mRNA in the dorsolateral prefrontal cortex in mice treated without or with LPS.

Then, we evaluated whether Akt inhibition could abrogate the preventive effect of KRIBB11 on LPS-induced depression-like behaviors (Figure 9A). For the immobility time in the TST, the two-way ANOVA showed significant effects for LPS injection (F_1,72_ = 20.86, *p* < 0.001), KRIBB11/LY294002/vehicle treatment (F_3,72_ = 4.08, *p* < 0.01), and the LPS × KRIBB11/LY294002/vehicle interaction (F_3,72_ = 3.36, *p* < 0.05) (Figure 9I). For the immobility time in the FST, the two-way ANOVA showed significant effects for LPS injection (F_1,72_ = 31.76, *p* < 0.001), KRIBB11/LY294002/vehicle treatment (F_3,72_ = 3.36, *p* < 0.05), and the LPS × KRIBB11/LY294002/vehicle interaction (F_3,72_ = 4.35, *p* < 0.01) (Figure 9J). Post hoc analysis revealed that the intracerebroventricular LY294002 infusion abrogated the preventive effect of KRIBB11 on LPS-induced increases in the immobility time in the TST (Figure 9I) and FST (Figure 9J). Taken together, our results demonstrated that Akt inhibition can abrogate the preventive effect of KRIBB11 on LPS-induced neuroinflammatory responses and depression-like behaviors.

## Discussion

One of the major contributions of the present study was the identification of a novel compound that induces microglial process elongation KRIBB11. The KRIBB11 incubation was found to induce a dramatic elongation of the processes in the primary cultured microglia in a dose- and time-dependent manner. The microglia in the dorsolateral prefrontal cortex in mice administered with KRIBB11 also displayed elongated processes, suggesting that the pro-elongation effect of KRIBB11 on microglial process can occur at conditions *in vivo*. We also observed that the pro-elongation effect of KRIBB11 on microglial process at conditions *in vitro* and *in vivo* disappeared along with KRIBB11 washout or the discontinuation of KRIBB11 treatment, which was consonant with the plasticity nature of microglia (Yang R. et al., 2019) and indicated that the pro-elongation effect of KRIBB11 on microglial processes is reversible.

The high ramified processes in microglia are important structures that can respond to the surrounding environment and pathological damages in the developing and adult brain. The microglia utilize their ramified processes to exert various functions such as synaptic pruning, synapse remodeling, and debris clearance (Harry, 2013; Bar and Barak, 2019; Gosselin, 2020), and the abnormal retraction of their ramified processes would disrupt brain homeostasis and even promote or mediate the development of central nervous system disorders, which has been widely confirmed by previous studies (Giunti et al., 2014; Davies et al., 2017; Hanlon et al., 2019; Matta et al., 2020). Thus, using drugs that make the retracted processes in microglia return into a ramified state would help to develop novel strategies for the prevention or treatment of central nervous system disorders. The present findings may provide a potential candidate for that purpose, as KRIBB11 was not only found to induce elongations of microglial process at physiological conditions, but also prevent the LPS-induced retractions of microglial processes at conditions *in vitro* and *in vivo*, which represented an activated state.

The pathologically activated microglia usually display dynamic changes in morphology and cell functions, such as the shortening of the ramified process, the overproduction of pro-inflammatory cytokines, and the impairment of the anti-inflammatory ability. The shortening of microglia process is positively associated with the progression neuroinflammatory response (Reichert and Rotshenker, 2019; Takagi et al., 2019), while the elongation of microglia process produces an anti-neuroinflammatory phenotype (Vinet et al., 2012; Yang R. et al., 2019). Suppression of the neuroinflammatory response by methods such as the supplementation of β-hydroxybutyrate (Huang et al., 2018), butyrate (Wang et al., 2018), diallyl disulfide (Xu et al., 2020), and sulforaphane (Wu et al., 2019) or the use of mesenchymal stem cells (Neubrand et al., 2014), astrocyte-conditioned media (Liu et al., 1994), epithelial cells-conditioned media (Wilms et al., 1997) have been shown to skew the primary cultured microglia toward a morphology phenotype with elongated processes. Our findings showed that the KRIBB11 incubation not only promoted microglial process elongation but also induced remarkable increases in the gene expression levels of anti-inflammatory mediators such as IL-4, IL-10, arginase-1, and CD206 in primary cultured microglia and dorsolateral prefrontal cortexes, suggesting that the KRIBB11 may have neuroprotective functions at physiological conditions. Our results also showed that the KRIBB11 pretreatment prevented the LPS-induced increases in the expression levels of IL-1β, IL-6, and TNF-α mRNA as well as the LPS-induced decreases in the expression levels of IL-4, IL-10, arginase-1, and CD206 mRNA in primary cultured microglia and dorsolateral prefrontal cortexes. These findings, together with a previous finding that the elongation of macrophage process by shaping macrophage morphology directly induces an anti-inflammatory phenotype (Neubrand et al., 2014), provide further evidence to establish the correlations between microglial process elongation and anti-neuroinflammation. The over-production of pro-inflammatory mediators indicates an M1 polarization state, and the increase in anti-inflammatory mediators shows an M2 polarization state (Tang and Le, 2016). The M1 and M2 polarization state are functionally interconnected, as the M2 polarization mediators, such as the IL-4, can promote anti-inflammation and debris clearance (Yang et al., 2019a; Yang et al., 2019b). In the present study, although the KRIBB11 treatment induced a remarkable increase in the expression levels of IL-4, at the present stage we still do not know whether it is the increased IL-4 that mediates the inhibitory effect of KRIBB11 on neuroinflammation in LPS-stimulated microglia and dorsolateral prefrontal cortexes.

Our functional studies also showed that the 5 days of KRIBB11 treatment almost completely prevented the LPS-induced depression-like behaviors in the TST and FST in mice, suggesting that the KRIBB11 may have an ability to ameliorate depressive symptoms in animal models of depression and could be developed as a novel drug for depression treatment. However, as the herein-used model of depression was induced by LPS treatment, and the mostly-used models of depression was usually induced by some other types of chronic stresses, such as the chronic social defeat stress (Wang W. et al., 2021) and chronic unpredictable stress (Strekalova et al., 2022), the above-mentioned hypotheses should be investigated carefully in future studies by using the other models of depression. Furthermore, as our results showed that the KRIBB11 treatment prevented the development of the M1 phenotype-associated pro-inflammatory responses in the brain in LPS-challenged mice, we suppose that the KRIBB11 may also have therapeutic values in the other central nervous system disorders associated with neuroinflammation, such as Alzheimer’s disease (Uddin et al., 2022) and Parkinson’s disease (Badanjak et al., 2021). Another limitation that should be pointed out is that we only presented the pro-elongation effect of KRIBB11 on microglia process at conditions *in vivo* in the dorsolateral prefrontal cortex. In fact, the pro-elongation effect of KRIBB11 on microglia process at conditions *in vivo* could be also observed in other regions of the brain such as the hippocampus (data not shown). As neuroinflammation in different regions of the brain such as the prefrontal cortex, hippocampus, and amygdala are highly associated with the pathogenesis of depression, we hypothesize that the herein-observed antidepressant-like effects of KRIBB11 may be due to its effects on microglial process and neuroinflammatory responses in different regions of the brain. However, this hypothesis should be examined by future studies.

The elongation of microglial process induced by compounds, such as β-hydroxybutyrate (Huang et al., 2018), butyrate (Wang et al., 2018), diallyl disulfide (Xu et al., 2020), and sulforaphane (Wu et al., 2019), has been shown to be mediated by Akt activation. We showed here that the Akt activation may be also required for the pro-elongation effect of KRIBB11 on microglial process, as 1) KRIBB11 incubation can induce a significant increase in the phosphorylation levels of Akt in the primary cultured microglia, and 2) Akt inhibition can abrogate the pro-elongation effect of KRIBB11 on microglial process at conditions *in vitro* and *in vivo*. If the pro-elongation effect of KRIBB11 on microglia process was suppressed by Akt inhibition, the regulatory effects of KRIBB11 on neuroinflammatory response and neuroinflammation-associated behavioral abnormalities would also be suppressed. As anticipated, inhibition of Akt by LY294002, a classical of the Akt signal, abrogated the preventive effect of KRIBB11 on LPS-induced increases in the expression levels of IL-1β, IL-6, and TNF-α mRNA as well as LPS-induced decreases in the expression levels of IL-4, IL-10, arginase-1, and CD206 mRNA in primary cultured microglia and dorsolateral prefrontal cortexes. Furthermore, the Akt inhibition was found to abrogate the preventive effect of KRIBB11 on LPS-induced depression-like behaviors in the TST and FST. These results may also help to establish a causal correlation between the pro-elongation of KRIBB11 on microglial process and the anti-neuroinflammatory effect of KRIBB11. A major limitation for the present findings is that we did not check the changes in the phosphorylation levels of Akt in the primary cultured microglia and the dorsolateral prefrontal cortexes treated with or without LPS and/or LY294002. In future studies, we would check the role of the Akt signal in pro-inflammatory stimuli (such as LPS)-induced changes in microglial morphology and inflammatory responses.

How exactly KRIBB11 induces microglial process elongation remains unclear. KRIBB11 is initially identified as an inhibitor of HSF1 (Yoon et al., 2011). Our previous studies had reported that the inhibition of HSF1 by KRIBB11 can prevent the expression and production of iNOS and NO in primary cultured microglia and brain tissues (Huang et al., 2015). Thus, we suppose that the HSF1 inhibition may be a core mechanism that mediates the pro-elongation effect of KRIBB11 on microglial process. However, through analysis of previously published literatures, we found no evidence that can support the direct involvement of HSF1 in microglial process regulation, and no evidence could be available to support the regulation of Akt by HSF1. By contrary, it is the HSF1 that has been widely reported to be regulated by Akt (Schulz et al., 2014; Carpenter et al., 2015; Frezzato et al., 2019; Tang et al., 2020). For example, Tang et al. had reported that the Akt can enhance the heat shock response in human embryonic kidney (HEK) 293T cells by inducing the phosphorylation of HSF1 at Ser230 (Tang et al., 2020). High levels of heat shock protein 70 (Hsp70) in chronic lymphocytic leukemia cells were found to express high levels of phospho-Akt at Ser473, thereby inducing a strong activation of HSF1 (Frezzato et al., 2019). In addition, the Akt has been reported to phosphorylate and activate HSF1 independently of heat-shock, leading to epithelial-mesenchymal transition in breast cancer cells (Schulz et al., 2014; Carpenter et al., 2015). Thus, we hypothesize that the overactivated HSF1 triggered by Akt may in turn reduce Akt activity as a negative feedback mechanism, through which the HSF1 inhibition may induce Akt activation and promote microglial process elongation.

Some early studies have also reported that the Akt activation relies on the Gi protein signal pathway (Kim et al., 2004), and the Gi signal activation in microglia is capable of inducing microglial process elongation and chemotaxis (Eyo et al., 2015; Bernier et al., 2019). Therefore, the signal that is associated with Gi activation may also mediate the pro-elongation effect of KRIBB11 on microglial process. This hypothesis could be also evidenced by a previous finding that the activation of the complement C3a receptor, a Gi-coupled receptor, in microglia can promote microglial process chemotaxis (Chen et al., 2020). It is known that the 5-hydroxyteyptamine (5-HT) is a key molecule that mediates the antidepressant actions of many clinically available antidepressants, such as sertraline and paroxetine (Furukawa et al., 2019). In a previous study by Etienne et al. (2019), researchers have found that a local application of 5-HT in acute brain slices can induce microglia process elongation, suggesting that the promotion of an anti-neuroinflammation-associated elongation of microglial process may be correlated with the antidepressant effects of 5-HT. Here, considering that the KRIBB11 pretreatment prevented the LPS-induced depression-like behaviors in mice, the KRIBB11 may induce microglial process elongation by promoting the release of 5-HT in microglia. Generally, all of these hypotheses provide more possible insight into the molecular mechanisms underlying the regulatory effect of KRIBB11 on microglia process, which should be investigated carefully in future studies, such as by using the proteomics technique to identify the intracellular signal pathway that mediates the pro-elongation effect of KRIBB11 on microglial process.

## Conclusion

Our results showed that the KRIBB11 can induce microglial process elongation at conditions *in vitro* and *in vivo*, which is correlated with anti-neuroinflammation. As this is the first report showing the regulatory effect of KRIBB11 on microglial process, this finding may provide an opportunity to develop novel mechanisms-based drugs for the treatment of central nervous system disorders associated with neuroinflammation. In future studies, in-depth studies should be conducted to explore more pharmacological characteristics of KRIBB11 in the central nervous system. For instance, we should investigate whether it has an ability to penetrate the blood-brain-barrier and whether it produces toxic effects when it is given at a high dosage. Furthermore, as we had previously identified a series of compounds that promote microglial process elongation, such as β-hydroxybutyrate (Huang et al., 2018), butyrate (Wang et al., 2018), diallyl disulfide (Xu et al., 2020), and sulforaphane (Wu et al., 2019), we should further compare the properties between KRIBB11 and those compounds on microglial process regulation, which may help to investigate the exact mechanisms underlying the pro-elongation effect of KRIBB11 on microglial process. For example, as the histone deacetylase (HDAC) inhibitor (such as trichostatin A) has been reported to promote microglial process elongation (Huang et al., 2018), and the β-hydroxybutyrate, butyrate, diallyl disulfide, and sulforaphane (Myzak and Dashwood, 2006; Shimazu et al., 2013; Huang et al., 2018) can suppress the activities of HDACs, it is reasonable to speculate that the KRIBB11 may have an ability to inhibit HDAC activities.

## Data Availability

The original contributions presented in the study are included in the article/Supplementary Material, further inquiries can be directed to the corresponding authors.
